# Effect of ligustrazine on preventing contrast-induced nephropathy in patients with unstable angina

**DOI:** 10.18632/oncotarget.21310

**Published:** 2017-09-28

**Authors:** Ziliang Ye, Haili Lu, Qiang Su, Xinhua Xian, Lang Li

**Affiliations:** ^1^ Department of Cardiology, The First Affiliated Hospital of Guangxi Medical University, Guangxi Cardiovascular Institute, Nanning, Guangxi, China; ^2^ Guangxi Medical University, Nanning, Guangxi, China

**Keywords:** ligustrazine, contrast-induced nephropathy, unstable angina

## Abstract

**Objective:**

Our purpose was to assess the effect of ligustrazine in the prevention of contrast-induced nephropathy (CIN) in patients with unstable angina (UA).

**Methods:**

148 patients with UA undergoing coronary angiography and/or percutaneous coronary intervention (PCI) were selected for observation; the patients were divided into a control group (group A, n=74) and a ligustrazine group (group B, n=74). Group A was given routine treatment, while group B was given routine treatment combined with ligustrazine. Serum creatinine (Scr), cystatin C and glomerular filtration rate (eGFR) concentrations were measured before and 1 day, 2 days and 3 days after treatment, and the incidence of contrast-induced nephropathy (CIN) and major cardiovascular events (MACE) were observed in both groups.

**Results:**

The Scr, Cystatin C and eGRF levels in group B were better than in group A after 1 day (OR: 2.64, 95% CI: 2.47-4.98; OR: 2.66, 95% CI: 2.62-5.77; OR: 4.02, 95% CI: 3.02-5.53, respectively), 2 days (OR: 3.58, 95% CI: 2.41-4.92; OR: 2.92, 95% CI: 2.83-5.02; OR: 3.28, 95% CI: 3.24-5.14, respectively) and 3 days of treatment (OR: 3.26, 95% CI: 2.17-4.35; OR: 2.85, 95% CI: 2.26-4.02; OR: 3.19, 95% CI: 2.53-4.34, respectively). The incidence of CIN (9.26% vs 16.67%) and MACE (7.41% vs 18.51%) of group B were significantly lower than in group A (P<0.05).

**Conclusions:**

Our study suggests that ligustrazine can reduce CIN and MACE in patients with UA when undergoing coronary angiography and/or PCI.

## INTRODUCTION

Contrast-induced nephropathy (CIN) [1, 2, 3] is an acute renal function injury that occurs within 3 days after using contrast agent when excluding other factors that may damage renal function. In hospital-acquired acute kidney injury, CIN has become the third major cause of renal perfusion decrease and renal drug toxicity [4, 5]. At present, CIN is defined as an impairment of renal function determined by either a 25% increase in SCr from baseline or a 0.5 mg/dL increase in absolute value within 48 to 72 hours of intravenous contrast administration [6, 7]. Previous research results have shown that in patients who receive contrast agents, the incidence of CIN is approximately 1%-6% [8]. Once CIN has occurred, hospitalization time is longer, and the cost of health care increases, presenting a considerable economic burden to society. Because no effective measure for the treatment of CIN has been developed, research for CIN is primarily focused on prevention.

At present, rehydration therapy is the most respected clinical guidelines for the prevention of CIN. European and American coronary intervention guidelines recommend that patients with chronic kidney disease undergoing cardiac catheterization should be fully prepared for the prevention of CIN [9, 10]. In recent years, many studies have noted that a variety of drugs can be used to prevent CIN, such as N-acetylcysteine (NAC) [11], theophylline [12, 13], vitamin C [14, 15], statins [16, 17] and prostaglandin [18, 19]. Nevertheless, the efficacy of these drugs remains controversial.

At present, the molecular mechanism of CIN has not been thoroughly elucidated to date [20]. It is generally believed that the direct toxic effects and the influence of the renal hemodynamics of contrast agent on renal tubules may play an important role in CIN [21, 22, 23]. In recent years, the apoptosis of renal tubular cells induced by contrast agents, which is an important mechanism of CIN, has attracted increasing attention and is considered to be one of the most important causes of CIN. Contrast agent can lead to excessive apoptosis of renal tubular epithelial cells, which can cause the damage of renal tubular cells. Studies have highlighted several possible mechanisms of renal tubular apoptosis induced by contrast agents, including Caspase-3 activation and Ca^2+^ overload, in renal tubular cells [24, 25].

Ligustrazine [26, 27], also known as natural four methyl, can not only expand peripheral blood vessels and inhibit platelet aggregation effect, but also influences the clearing of free radicals, the scavenging of reactive oxygen species, and the blocking of calcium channels [28]. Gong [29] found that ligustrazine can be used to prevent acute kidney injury caused by CIN, and that this effect may be mediated by inhibition of p38MAPK and FoxO1-mediated signal transduction pathways, reducing the apoptosis of renal tubular epithelial cells.

Several basic science experiments have been conducted [30, 31] to verify that ligustrazine prevents CIN by inhibiting the apoptosis of renal tubular epithelial cells, but the effect of ligustrazine on preventing CIN still lacks clinical evidence. Therefore, the purpose of our study was to evaluate the clinical effect of ligustrazine in the prevention of CIN in patients with UA and to provide clinical evidence for the prevention of CIN.

## RESULTS

### Patient population and baseline characteristics

After excluding the patients who did not meet the diagnosis of UA and those patients who were not suitable for this study, a total of 148 patients aged 56-68 years [the mean (SD) age was 62.73 (7.3) years] were enrolled in our study; 87 (58.78%) patients were male. Smoking, previous MI, albumin, HDL-C, iodixanol dosage, ACEIs/ARBs, beta-blockers and GPIIb/IIIa inhibitors were not similar between group A and group B (all P <0.05). However, age, male sex, BMI, hypertension, diabetes mellitus, hyperlipidaemia, previous CABG, LVEF, TC, LDL-C, calcium antagonists and nitrates were similar between group A and group B (all P >0.05). The patients’ population statistics and clinical characteristics are presented in Table 1.

**Table 1:** Baseline characteristics of the patients

Characteristic	Group A (n=74)	Group B (n=74)	P value
Age (year)	62.35±7.37	63.08±7.29	0.5456
Male sex, n (%)	42(56.75)	45(60.81)	0.616
BMI(kg/m^2^)	23.6±2.5	24.2±3.5	0.2321
Hypertension, n (%)	26(35.13)	32(43.24)	0.312
Diabetes mellitus, n (%)	15(20.27)	25(33.78)	0.064
Smoking, n (%)	37(50.00)	49(58.33)	0.046
Hyperlipidaemia, n (%)	32(43.24)	21(28.37)	0.059
Previous CABG, n (%)	3(4.05)	8(10.81)	0.117
Previous MI, n (%)	17(22.97)	6(8.10)	0.013
LVEF, n(%)	55.39±8.04	54.46±7.89	0.4787
Albumin (g/L)	34.23±6.39	37.36±7.03	0.0052
TC (mg/dL)	172.03±14.28	173.29±12.98	0.5752
HDL-C (mg/dL)	34.55±5.32	36.47±6.35	0.0480
LDL-C (mg/dL)	106.46±12.39	109.77±14.34	0.1351
Scr before treatment (umol/L)	98.23±12.73	96.79±13.47	0.5050
cystatin C before treatment (mg/L)	1.06±0.25	1.01±0.22	0.1985
eGFR before treatment (ml/min)	82.25±8.16	84.92±8.79	0.0574
Renal insufficiency, n (%)	0(0)	0(0)	NS
Iodixanol dosage (ml)	125.32±10.28	129.47±11.34	0.0210
Treatment received			
Aspirin, n (%)	74(100)	74(100)	NS
Clopidogrel sulfate, n (%)	74(100)	74(100)	NS
ACEIs/ARBs, n (%)	62(83.78)	51(68.91)	0.033
Beta-blockers, n (%)	67(90.54)	54(72.97)	0.006
Calcium antagonists, n (%)	31(41.89)	26(35.13)	0.398
Nitrates, n (%)	39(52.70)	31(41.89)	0.188
GPIIb/IIIa inhibitors, n (%)	26(35.13)	9(12.16)	0.001

Data are presented as number (percent) or mean±SD.

BMI=Body mass index; CABG=coronary artery bypass grafting, MI=myocardial infarction, LVEF=Left ventricular ejection fraction; TC=Total cholesterol; HDL-C=High density lipoprotein; LDL-C=Low density lipoprotein; Scr=serum creatinine; eGFR=glomerular filtration rate; ACEIs=Angiotensin-converting enzyme inhibitors; ARBs=Angiotensin-receptor blockers; GPIIb/IIIa inhibitors=Glycoprotein IIb/IIIa inhibitors; NS=Not significant.

### Scr, Cystatin C and eGRF levels after 1 day of treatment

Table 2 shows the levels of Scr, Cystatin C and eGRF after 1 day of treatment. After adjusting for smoking, previous MI, albumin, HDL-C, iodixanol dosage, ACEIs/ARBs, beta-blockers and GPIIb/IIIa inhibitors, the Scr, Cystatin C and eGRF levels in group B were better than in group A after 1 day of treatment (OR: 2.64, 95% CI: 2.47-4.98; OR: 2.66, 95% CI: 2.62-5.77; OR: 4.02, 95% CI: 3.02-5.53, respectively).

**Table 2:** Scr, Cystatin C and eGRF levels after 1 day of treatment

	Male	Female	Total
Scr at 1 day			
group A	0	0	0
group B	2.63 (1.51, 3.83) 0.041	2.79 (2.42, 4.77) 0.033	2.64 (2.47, 4.98) 0.037
Cystatin C at 1 day			
group A	0	0	0
group B	2.57 (2.26, 5.76) 0.041	2.99 (2.43, 4.77) 0.009	2.66 (2.62, 5.77) 0.022
eGRF at 1 day			
group A	0	0	0
group B	4.25 (2.03, 5.92) 0.030	3.06 (2.38, 4.44) 0.000	4.02 (3.02, 5.53) 0.037

Results are presented as OR (95% CI) P value.

Adjust for: smoking, previous MI, albumin, HDL-C, iodixanol dosage, ACEIs/ARBs, beta-blockers and GPIIb/IIIa inhibitors.

MI=myocardial infarction, HDL-C=High density lipoprotein; ACEIs=Angiotensin-converting enzyme inhibitors; ARBs=Angiotensin-receptor blockers; GPIIb/IIIa inhibitors=Glycoprotein IIb/IIIa inhibitors; NS=Not significant.

Scr, Cystatin C and eGRF levels after 2 days of treatment Table 3 shows the levels of Scr, Cystatin C and eGRF after 2 days of treatment. After adjusting for smoking, previous MI, albumin, HDL-C, iodixanol dosage, ACEIs/ARBs, beta-blockers and GPIIb/IIIa inhibitors, the Scr, Cystatin C and eGRF levels in group B were better than in group A after 2 days of treatment (OR: 3.58, 95% CI: 2.41-4.92; OR: 2.92, 95% CI: 2.83-5.02; OR: 3.28, 95% CI: 3.24-5.14, respectively).

**Table 3:** Scr, Cystatin C and eGRF levels after 2 days of treatment

	Male	Female	Total
Scr at 2 day			
group A	0	0	0
group B	3.63 (2.45, 3.89) 0.023	3.53(2.58, 4.89) 0.029	3.58 (2.41, 4.92) 0.031
Cystatin C at 2 day			
group A	0	0	0
group B	2.87 (2.26, 5.82) 0.014	3.69 (2.73, 4.98) 0.000	2.92 (2.83, 5.02) 0.026
eGRF at 2 day			
group A	0	0	0
group B	3.67 (2.23, 5.42) 0.003	3.14 (2.21, 4.28) 0.017	3.28 (3.24, 5.14) 0.043

Results are presented as OR (95% CI) P value.

Adjust for: smoking, previous MI, albumin, HDL-C, iodixanol dosage, ACEIs/ARBs, beta-blockers and GPIIb/IIIa inhibitors.

MI=myocardial infarction, HDL-C=High density lipoprotein; ACEIs=Angiotensin-converting enzyme inhibitors; ARBs=Angiotensin-receptor blockers; GPIIb/IIIa inhibitors=Glycoprotein IIb/IIIa inhibitors; NS=Not significant.

### Scr, Cystatin C and eGRF levels after 3 days of treatment

Table 4 shows the levels of Scr, Cystatin C and eGRF after 3 days of treatment. After adjusting for smoking, previous MI, albumin, HDL-C, iodixanol dosage, ACEIs/ARBs, beta-blockers and GPIIb/IIIa inhibitors, the Scr, Cystatin C and eGRF levels in group B were better than in group A after 3 days of treatment (OR: 3.26, 95% CI: 2.17-4.35; OR: 2.85, 95% CI: 2.26-4.02; OR: 3.19, 95% CI: 2.53-4.34, respectively).

**Table 4:** Scr, Cystatin C and eGRF levels after 3 days of treatment

	Male	Female	Total
Scr at 3 day			
group A	0	0	0
group B	3.02 (2.24, 3.18) 0.000	3.75(2.35, 4.92) 0.000	3.26 (2.17, 4.35) 0.013
Cystatin C at 3 day			
group A	0	0	0
group B	2.81 (2.02, 4.81) 0.043	3.03 (2.35, 4.36) 0.003	2.85 (2.26,4.02) 0.047
eGRF at 3 day			
group A	0	0	0
group B	3.55 (2.58, 5.02) 0.000	3.04 (2.78, 3.98) 0.023	3.19 (2.53, 4.34) 0.027

Results are presented as OR (95% CI) P value.

Adjust for: smoking, previous MI, albumin, HDL-C, iodixanol dosage, ACEIs/ARBs, beta-blockers and GPIIb/IIIa inhibitors.

MI=myocardial infarction, HDL-C=High density lipoprotein; ACEIs=Angiotensin-converting enzyme inhibitors; ARBs=Angiotensin-receptor blockers; GPIIb/IIIa inhibitors=Glycoprotein IIb/IIIa inhibitors; NS=Not significant.

### Incidence of CIN and MACE

Overall, CIN developed in 16.21% of all patients (24/148), and the prevalence of CIN was 20.27% (15/74) and 12.16% (9/74) in group A and group B, respectively, P<0.05. Furthermore, MACE developed in 10.81% of all patients (16/148), while the prevalence of MACE was 14.86% (11/74) and 6.75% (5/74) in group A and group B, respectively, P<0.05.

## DISCUSSION

In our study, we found that ligustrazine significantly reduced the levels of Scr and cystatin C in patients with UA after coronary angiography and/or PCI, and improved the level of eGFR after operation. Our results were in agreement with Gong X [29] and Cao S [32]. In addition, our results show that after ligustrazine administration, the incidence of CIN in group B was significantly lower than in group A (12.16% vs. 20.27%, P<0.05). Moreover, the incidence of MACE in group B was significantly lower than in group A, and that the difference was statistically significant (6.75% vs. 14.86%, P<0.05).

With the continuous development of PCI, the incidence of CIN is increasing year by year and has become the third-largest cause of kidney damage. In recent years, animal studies [31, 32] have found that ligustrazine effectively prevented the occurrence of CIN. However, because most of these studies were in animals, the results cannot automatically be generalized to humans. Furthermore, the research about ligustrazine in the prevention of CIN in the human body is scarce providing little theoretical reference. Therefore, we designed and carried out this study to fill that gap in knowledge. Indeed, our results show that ligustrazine prevented the occurrence of CIN in patients with UA after angiography and/or PCI, and reduced the incidence of MACE. Gong X [29] observed the effect of ligustrazine in the prevention of CIN in a rat model. Scr, blood urea nitrogen, cystatin C, urinary N-acetyl-β-glucosaminidase and urinary-glutamyl transpeptidase were tested to assess the kidney function, and the results demonstrate that ligustrazine significantly improved nephritic function and reduced the apoptosis of nephritic tubules in rats. Based on these data, the researchers concluded that ligustrazine could be an effective drug to prevent CIN in the future. Gong SZ [33] also studied the effect of ligustrazine in the prevention of CIN in rat model. Twenty-four rats were randomly divided into a normal group (n=8), a model group (n=8) and a ligustrazine group (n=8). SCr, blood urea nitrogen (BUN) and Cystatin C were tested 24 h after the CIN model induction; meanwhile, the morphology of the kidney and the number of apoptotic cells were also observed. 24 h after modeling, the levels of Scr, BUN, cystatin C in the model group were significantly higher than in the normal group, demonstrating significant nephritic injury (P<0.01). Compared to the model group, the Scr, BUN and cystatin C levels of the ligustrazine group were significantly decreased (P<0.01). In addition, compared to the model group, the nephritic interstitial lesions in the ligustrazine group were significantly lightened. The morphology and structure were relatively intact, and the area of nephritic tubular epithelial cells was significantly decreased. Furthermore, the apoptosis of nephritic cells in the ligustrazine group was significantly decreased, as was the apoptosis index, in comparison to the model group (all P<0.01).

At present, the mechanism by which ligustrazine prevents CIN is unclear but may be related to the following factors. 1) ligustrazine increases the expression of the anti-apoptotic protein Bcl-2 in renal tubular epithelial cells, reduces the expression of the pro-apoptotic bax protein, and reduces the apoptosis of renal tubular epithelial cells induced by contrast agents [34]. Meanwhile, ligustrazine reduces the degree of the degeneration of renal tubular epithelial cells induced by contrast agents, preventing damage to the cells and the resultant decrease of renal function [35]. 2) ligustrazine expands renal blood vessels, increases renal blood flow, reduces ischemia of the renal medulla, and later reduces the hypoxia and oxidative stress in the renal medulla induced by contrast agent, thereby preventing CIN [36]. 3) The latest findings suggest that p38 MAPK pathways play an important role in CIN [29]. P38 MAPK signaling participates in the apoptosis process and is important for renal tubular cell apoptosis. The activation of p38 MAPK is associated with iNOS induction and decreases the expression of Bcl-2, which is a protein that plays a vital role in anti-apoptosis. Ligustrazine inhibits signaling through the p38 MAPK pathway, thereby decreasing the total number of apoptotic cells [37].

### Limitations

Our research was limited by several important 1) At present, there is little clinical research regarding the effect of ligustrazine on the prevention of CIN in patients with UA, and our study was conducted in a single center; therefore, our results may not extend to other populations. 2) In this study, the participants were mainly aged (aged 56-68 years), and the renal function was normal. However, in many cases, patients who need med coronary angiography or PCI are often associated with renal impairment. When using ligustrazine, whether or not patient’s kidney function will become more serious is unknown and will require further clinical trials in the future. 3) The primary ingredient of ligustrazine is extracted from a traditional Chinese medicine. The efficacy and safety have not been recognized by North American and European countries. Therefore, a degree of confounding bias may exist. 4) As we can see from the Table 1, the use of GP IIb/IIIa inhibitors in the group A was higher than of the group B, this phenomenon indirect means that GP IIb/IIIa inhibitors may have an influence on cardiac condition and/or kidney function, and then increases the rate of MACE in group A, despite a regression analysis was conducted to adjust potential confounding factors (such as GP IIb/IIIa inhibitors and other medications).

## MATERIALS AND METHODS

### Patients and methods

#### Study population

We conducted this study at a single hospital center in the First Affiliated Hospital of Guangxi Medical University from October 2014 to October 2016, and our research model obtained the approval of the Ethics Committee of the First Affiliated Hospital of Guangxi Medical University.

**Figure 1:**
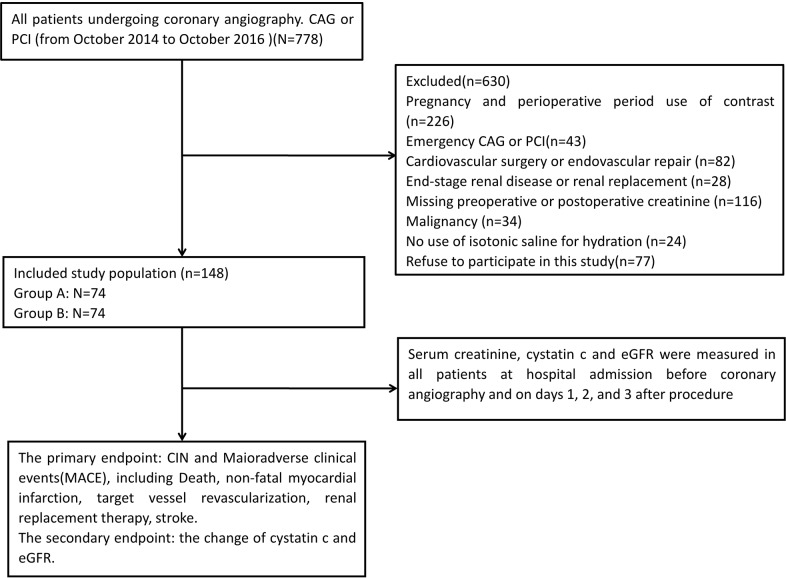
Flow diagram of patients with unstable angina undergoing coronary angiography and/or PCI.

First, 778 patients undergoing coronary angiography and/or percutaneous coronary intervention (PCI) in our hospital from October 2014 to October 2016 were selected for observation. Six hundred thirty patients were excluded from our study for pregnancy and perioperative period use of contrast (n=226), emergency CAG or PCI (n=43), cardiovascular surgery or endovascular repair (n=82), end-stage renal disease or renal replacement (n=28), missing preoperative or postoperative creatinine (n=116), malignancy (n=34), no use of isotonic saline for hydration (n=24), or refusal to participate (n=77). Finally, 148 patients diagnosed with UA were enrolled in the study. The flow diagram of patients with unstable angina undergoing coronary angiography or PCI is shown as Figure 1. Written informed consent was obtained from all of the participants.

One hundred forty-eight patients were divided into the control group (group A, n=74) and the ligustrazine group (group B, n=74). At the same time, the general information about the patients was collected, such as age, sex, BMI, smoking habits, hypertension, and diabetes mellitus (other information can be found in Table 1). Upon enrollment, coronary angiography and/or PCI were performed according to the standard protocols and using standard techniques for coronary angiography and/or PCI, with the selection of surgical instruments and the experience of the surgeon also in accordance with the recommended guidelines. A non-ionic osmotic contrast agent (Iodixanol) was used; the choice to use a contrast agent was not limited and was guided by best practices for the required operation.

#### Medications

Group A was given routine treatment (physiological saline water was used from 12 h before until 12 h after the operation, 1 ml/kg/h, and aspirin, clopidogrel, and statins were given). Group B was given routine treatment combined with ligustrazine (from 3 days before the operation until 3 days after the operation, 120 mg/day, intravenous).

### Serum creatinine, cystatin C and glomerular filtration rate measurements

Venous blood samples were acquired at baseline (before the drug therapy and contrast agent were infused and before angiography or PCI) and at 1 day, 2 days and 3 days after the contrast agent was administered. The level of Scr was measured by the colorimetric method [12], while the level of cystatin C was measured by an immunonephelometric method [13]. Glomerular filtration rates (eGFR) were measured in two ways: eGFR (male)=186×(Cr/88.40)-1.154×age-0.203; or eGFR (female)=186×(Cr/88.4)-1.154×age-0.203×0.742, μmol/l [38, 25].

### End points and definitions

The changes in Scr, cystatin C and eGFR before and 1 day, 2 days and 3 days after the operation were observed. Meanwhile, the incidence of CIN and MACE was observed in the two groups. CIN is defined as the impairment of renal function determined by either a 25% increase in SCr from baseline or a 0.5 mg/dL increase in absolute value, within 48 to 72 hours of intravenous contrast administration. MACE was defined as a composite of cardiac death, non-fatal myocardial infarction (MI), stroke, target vessel revascularization (TVR), congestive heart disease, or ventricular arrhythmia.

### Statistical analysis

Continuous variables are presented as the means (±standard deviation; SD), categorical variables are presented as the frequency (percentage), and P-values <0.05 were considered statistically significant. The homogeneity of the two groups at baseline was evaluated with a chi-square test for categorical variables (such as gender, sex, hypertension, diabetes mellitus, smoking, hyperlipidemia, family history, previous CABG, previous MI and medications) and with ANOVA for continuous variables (such as age, BMI, LVEF, albumin, TC, HDL-C, LDL-C and iodixanol dosage). Finally, regression analysis was conducted to adjust for confounding factors. All of the statistical analyses were performed using SPSS 22.0 (SPSS Inc., Chicago, IL).

## CONCLUSIONS

The present study shows that ligustrazine reduced the incidence of CIN and MACE in patients with UA when undergoing coronary angiography and/or PCI. In the future, randomized, double-blind, placebo-controlled, parallel-group, multicenter research is warranted to confirm these results.

## References

[1] Toso A, Leoncini M, Maioli M, Tropeano F, Di VE Villani S, Bellandi F (2014). Relationship between inflammation and benefits of early high-dose rosuvastatin on contrast-induced nephropathy in patients with acute coronary syndrome: the pathophysiological link in the PRATO-ACS study (Protective Effect of Rosuvastatin and Antiplatelet. Jacc Cardiovascular Interventions.

[2] Golshahi J, Nasri H, Gharipour M (2014). Contrast-induced nephropathy; A literature review. Journal of Nephropathology.

[3] Mehran R, Aymong ED, Nikolsky E, Lasic Z, Iakovou I, Fahy M, Mintz GS, Lansky AJ, Moses JW, Stone GW (2004). A simple risk score for prediction of contrast-induced nephropathy after percutaneous coronary intervention: development and initial validation. Journal of the American College of Cardiology.

[4] He WU, Zhang F, Fei YE, You W, Mao J, Cardiology DO, Hospital C, Cardiology DO, Hospital NF, University NM (2015). Incidence and in-hospital outcomes of contrast-induced nephropathy based on different definitions in patients with acute coronary syndrome and relative normal renal function. Journal of Clinical Cardiology.

[5] Rosenstock JL, Gilles E, Geller AB, Panagopoulos G, Mathew S, Malieckal D, Devita MV, Michelis MF (2010). Impact of heart failure on the incidence of contrast-induced nephropathy in patients with chronic kidney disease. International Urology & Nephrology.

[6] Cronin RE (2010). Contrast-induced nephropathy: pathogenesis and prevention. Pediatric Nephrology.

[7] Azzalini L, Spagnoli V, Ly HQ (2016). Contrast-induced nephropathy: from pathophysiology to preventive strategies. Canadian Journal of Cardiology.

[8] Waybill MM, Waybill PN, Waybill MM, Waybill PN (2001). Re: contrast media-induced nephrotoxicity: identification of patients at risk and algorithms for prevention. Journal of Vascular & Interventional Radiology Jvir.

[9] Members ATF, Windecker S, Kolh P, Alfonso F, Collet JP, Cremer J, Falk V, Filippatos G, Hamm C, Head SJ (2015). 2014 ESC/EACTS Guidelines on myocardial revascularization. Pergamon Press.

[10] Members WC, Jneid H, Anderson JL, Wright RS, Adams CD, Bridges CR, Casey DE, Ettinger SM, Fesmire FM, Ganiats TG (2012). ACCF/AHA Focused update of the guideline for the management of patients with unstable Angina/Non–ST-Elevation myocardial infarction (Updating the 2007 guideline and replacing the 2011 focused update). Journal of the American College of Cardiology.

[11] Balderramo DC (2006). N-acetylcysteine and contrast-induced nephropathy. New England Journal of Medicine.

[12] Arabmomeni M, Najafian J, Abdar EM, Samadi M, Mirbagher L (2015). Comparison between theophylline, N-acetylcysteine, and theophylline plus N-acetylcysteine for the prevention of contrast-induced nephropathy. Arya Atherosclerosis.

[13] Ye Z, Lu H, Qiang S, Guo W, Dai W, Li H, Yang H, Lang L (2017). Clinical effect of trimetazidine on prevention of contrast-induced nephropathy in patients with renal insufficiency: An updated systematic review and meta-analysis. Medicine.

[14] Rollins KE, Noorani A, Janeckova L, Griffiths M, Baker MP, Boyle JR (2013). Vitamin C ameliorates renal injury in a murine model of contrast-induced nephropathy. Joint Meeting of the Section-of-Surgery of the Royal-Society-of-Medicine.

[15] Chen H (2011). Prevention of contrast-induced nephropathy with vitamin C in coronary heart disease patients with renal insufficiency. Journal of Clinical & Experimental Medicine.

[16] Chyou AC, Thodge A, Feldman DN, Swaminathan RV (2015). Statins in the Prevention of Contrast-Induced Nephropathy. Current Treatment Options in Cardiovascular Medicine.

[17] Peruzzi M, De LL Thomsen HS, Romagnoli E, D’Ascenzo F, Mancone M, Sardella G, Lucisano L, Abbate A, Frati G (2015). A network meta-analysis on randomized trials focusing on the preventive effect of statins on contrast-induced nephropathy. Biomed Res Int.

[18] Li WH, Li DY, Qian WH, Liu JL, Xu TD, Zhu H, He HY (2014). Prevention of contrast-induced nephropathy with prostaglandin E1 in high-risk patients undergoing percutaneous coronary intervention. International Urology & Nephrology.

[19] Kuan WU, Xi-Xiang YU, Lin YS (2012). The protection of prostaglandin E1 against contrast-induced nephropathy: an experimental study. Journal of Interventional Radiology.

[20] Katsiki N, Athyros VG, Karagiannis A, Mikhailidis DP (2015). Contrast-Induced Nephropathy: An “All or None” Phenomenon?. Angiology.

[21] Seeliger E, Flemming B, Wronski T, Ladwig M, Arakelyan K, Godes M, Möckel M, Persson PB (2007). Viscosity of contrast media perturbs renal hemodynamics. Journal of the American Society of Nephrology Jasn.

[22] Reddan D (2007). Patients at high risk of adverse events from intravenous contrast media after computed tomography examination. European Journal of Radiology.

[23] Bolognese L, Falsini G, Schwenke C, Grotti S, Limbruno U, Liistro F, Carrera A, Angioli P, Picchi A, Ducci K (2012). Impact of iso-osmolar versus low-osmolar contrast agents on contrast-induced nephropathy and tissue reperfusion in unselected patients with ST-segment elevation myocardial infarction undergoing primary percutaneous coronary intervention (from the Contrast. American Journal of Cardiology.

[24] Andreucci M, Faga T, Russo D, Bertucci B, Tamburrini O, Pisani A, Sabbatini M, Fuiano G, Michael A (2014). Differential activation of signaling pathways by low-osmolar and iso-osmolar radiocontrast agents in human renal tubular cells. Journal of Cellular Biochemistry.

[25] Quintavalle C, Brenca M, De MF Fiore D, Romano S, Romano MF, Apone F, Bianco A, Zabatta MA, Troncone G (2011). In vivo and in vitro assessment of pathways involved in contrast media-induced renal cells apoptosis. Cell Death & Disease.

[26] Kim M, Kim SO, Lee M, Lee JH, Jung WS, Moon SK, Kim YS, Cho KH, Ko CN, Lee EH (2014). Tetramethylpyrazine, a natural alkaloid, attenuates pro-inflammatory mediators induced by amyloid β and interferon-γ in rat brain microglia. European Journal of Pharmacology.

[27] Yan YX, Zhao JX, Han S, Zhou NJ, Jia ZQ, Yao SJ, Cao CL, Wang YL, Xu YN, Zhao J (2015). Tetramethylpyrazine induces SH-SY5Y cell differentiation toward the neuronal phenotype through activation of the PI3K/Akt/Sp1/TopoIIβ pathway. European Journal of Cell Biology.

[28] Sue YM, Cheng CF, Chang CC, Chou Y, Chen CH, Juan SH (2009). Antioxidation and anti-inflammation by haem oxygenase-1 contribute to protection by tetramethylpyrazine against gentamicin-induced apoptosis in murine renal tubular cells. Nephrology Dialysis Transplantation.

[29] Gong X, Wang Q, Tang X, Wang Y, Fu D, Lu H, Wang G, Norgren S (2013). Tetramethylpyrazine prevents contrast-induced nephropathy by inhibiting p38 MAPK and FoxO1 signaling pathways. American Journal of Nephrology.

[30] Yang QH, Liang Y, Xu Q, Zhang Y, Xiao L, Si LY (2011). Protective effect of tetramethylpyrazine isolated from Ligusticum chuanxiong on nephropathy in rats with streptozotocin-induced diabetes. Phytomedicine.

[31] Fu YJ, Zhou Y, Pan JQ, Zhang XM, Lü JH (2012). The rapeutic effects and mechanisms of tetramethylpyrazine on streptozocin-induced-nephropathy in type 2 diabetic rats. Journal of Chinese Pharmaceutical Sciences.

[32] Cao S, Zhao W, Bu H, Zhao Y, Yu C (2016). Ligustrazine for the treatment of unstable angina: a meta-analysis of 16 randomized controlled trials. Evi Based Comp Alt Med.

[33] Wang J, Zheng S, Xing H (2012). Experimental study on effect of ligustrazine on transdifferentiation of primary culture human embryo renal proximal epithelial cells induced by TGF-β_1. Chongqing Medicine.

[34] Xiao S, Li K, Lu H (2003). Effect of ligustrazine on the expression of Bcl-2 protein and apoptosis in rabbit articular chondrocytes in monolayer culture. Bulletin of Hunan Medical University.

[35] Xue Y, Tie CR, Li J, Tian T, Li QX (2011). Ligustrazine inhibits lipopolysaccharide-induced proliferation by affecting P27, Bcl-2 expression in rat mesangial cells. European Journal of Pharmacology.

[36] Yang QH, Liang Y, Xu Q, Zhang Y, Xiao L, Si LY (2011). Protective effect of tetramethylpyrazine isolated from Ligusticum chuanxiong on nephropathy in rats with streptozotocin-induced diabetes. Phytomedicine.

[37] Gong X, Celsi G, Carlsson K, Norgren S, Chen M (2010). N-acetylcysteine amide protects renal proximal tubular epithelial cells against iohexol-induced apoptosis by blocking p38 MAPK and iNOS signaling. American journal of nephrology.

[38] Romano G, Briguori C, Quintavalle C, Zanca C, Rivera NV, Colombo A, Condorelli G (2008). Contrast agents and renal cell apoptosis. European Heart Journal.

